# Climacocystaceae fam. nov. and Gloeoporellaceae fam. nov., two new families of Polyporales (Basidiomycota)

**DOI:** 10.3389/fmicb.2023.1115761

**Published:** 2023-02-03

**Authors:** Shun Liu, Jun-Liang Zhou, Jie Song, Yi-Fei Sun, Yu-Cheng Dai, Bao-Kai Cui

**Affiliations:** ^1^School of Ecology and Nature Conservation, Institute of Microbiology, Beijing Forestry University, Beijing, China; ^2^International Exchange and Cooperation Department, Kunming University, Kunming, Yunnan, China; ^3^Department of Horticulture and Food, Guangdong Eco-Engineering Polytechnic, Guangzhou, China

**Keywords:** molecular clock, multi-gene phylogeny, new family, taxonomy, white-rot fungi

## Abstract

Polyporales is a diverse group of Agaricomycetes including more than 2,500 species belonging to 255 genera and 18 families. Recently, many studies focused on the classification of Polyporales, but the familial placements of some taxa remain uncertain. In this study, two new families, Climacocystaceae and Gloeoporellaceae of Polyporales, are proposed based on morphological characters and molecular data. Phylogenetic analyses of the two new families are inferred from the DNA sequences of the internal transcribed spacer regions (ITS), the large subunit of nuclear ribosomal RNA gene (nLSU), the largest subunit of RNA polymerase II gene (RPB1), the second largest subunit of RNA polymerase II gene (RPB2), and the translation elongation factor 1-α gene (TEF1). Furthermore, the divergence time of Polyporales was estimated as an additional taxonomic criterion based on the conserved regions of five DNA fragments (5.8S, nLSU, RPB1, RPB2, and TEF1). Bayesian evolutionary analysis revealed that the ancestor of Polyporales splits with a mean stem age of 136.53 Mya with a 95% highest posterior density (HPD) of 118.08–158.06 Mya. The mean stem ages of the families within Polyporales originated between 66.02 and 119.22 Mya, of which Climacocystaceae occurred in a mean stem age of 77.49 Mya with a 95% HPD of 61.45–93.16 Mya, and Gloeoporellaceae occurred in a mean stem age of 88.06 Mya with a 95% HPD of 67.15–107.76 Mya.

## Introduction

Polyporales Gäum is one of the major orders of Basidiomycota (Kirk et al., 2008). Most species of the Polyporales are saprotrophic wood-decay fungi, which can cause white decay or brown decay of wood, and play a vital role in the degradation and reduction of forest ecosystems. Moreover, some species of Polyporales are edible fungi, medicinal fungi, or forest pathogens (Dai et al., 2007; Rajchenber and Robledo, 2013; Wu et al., 2019). Due to their important ecological functions and economic values, Polyporales had been extensively studied, and their members were increased rapidly. In Kirk et al. (2008), Polyporales contain about 1,800 species, 216 genera, and 13 families, while in He et al. (2019), about 2,500 species, 285 genera, and 18 families are included in Polyporales.

Previously, the establishment of families in Polyporales was basically based on morphological characteristics. Polyporaceae Fr. ex Corda is the oldest family in Polyporales, which was proposed by Fries (1838) to include all fungi with poroid hymenophores. Then, Irpicaceae Spirin and Zmitr., Meruliaceae Rea, Podoscyphaceae D.A. Reid, Sparassidaceae Herter, and Steccherinaceae Parmasto were proposed successively and still legitimately exist in the current concept of Polyporales (Herter, 1910; Rea, 1922; Reid, 1965; Parmasto, 1968; Spirin, 2003; Justo et al., 2017). Jülich (1981) proposed a considerable number of families of Basidiomycetes, some of which belong to Polyporales were rarely used and treated as synonyms.

Since the 21st century, DNA sequencing and phylogenetic techniques have been widely used in the systematic study of Polyporales (Binder et al., 2005; Larsson, 2007; Miettinen et al., 2012). Binder et al. (2013) presented a phylogenetic and phylogenomic overview of the Polyporales and listed 40 validly published and legitimate family names. Zhao et al. (2015) introduced a new family Fragiliporiaceae Y-CD, B-KC, and C. L. Zhao based on the combination of morphological characters and molecular data. Justo et al. (2017) provided a phylogenetic overview of Polyporales, 18 clades in the Polyporales were assigned at the family level; the climacocystis clade (*Climacocystis* Kotl. and Pouzar, *Diplomitoporus* Domański) and *Tyromyces merulinus* (Berk.) G. Cunn. cannot be assigned to a family within Polyporales. He et al. (2019) carried out an outline of all genera of Basidiomycota; in which 19 families were placed in Polyporales, including 18 families accepted by Justo et al. (2017) and Fragiliporiaceae. However, the genera *Climacocystis* and *Diplomitoporus* remained with an uncertain familial placement. Liu et al. (2022) presented a systematic classification and phylogenetic relationships of the brown-rot fungi within the Polyporales; the study showed that 29 clades are assigned a family name, including four new brown-rot fungal families, *viz*., Auriporiaceae B.K. Cui, Shun Liu & Y.C. Dai, Piptoporellaceae B.K. Cui, Shun Liu & Y.C. Dai, Postiaceae B.K. Cui, Shun Liu & Y.C. Dai and Taiwanofungaceae B.K. Cui, Shun Liu & Y.C. Dai. They focused on the phylogenetic relationships of the brown-rot fungi within the Polyporales, and the number and composition of white-rot fungi family were consistent with the study of He et al. (2019).

Fruiting body types and wood decay types are two key traits in the evolutionary origins and genetic bases of fungi (Nagy et al., 2017). The Polyporales is a diverse group of Agaricomycetes, not only in molecular sequences but also in morphological characteristics (fruiting body: Resupinate, effused-reflexed, pileate-sessile, pileate-stipitate, cauliflower-like, etc.; Hymenophores: Poroid, daedaleoid, hydnoid, lamellate, labyrinthine, odontoid, etc.). Moreover, Polyporales include two types of wood decay fungi, white-rot fungi and brown-rot fungi (Binder et al., 2013; Justo et al., 2017). The varied fruiting body types and wood decay types indicate that there are complex evolutionary relationships among the members of Polyporales. Recently, divergence time was used as important criteria for the classification and estimation of evolutionary time in Basidiomycota (Chen et al., 2015; Song et al., 2016; Zhao et al., 2016, 2017; Song and Cui, 2017; He et al., 2019; Zhu et al., 2019; Wu et al., 2020; Wang et al., 2021; Ji et al., 2022).

During the investigations of wood decay fungi, abundant samples of *Climacocystis*, *Diplomitoporus*, and *Gloeoporellus* Zmitr. were collected. To determine their phylogenetic positions within Polyporales, phylogenetic analyses were carried out based on the combined sequence datasets of ITS + nLSU + RPB1 and ITS + nLSU + RPB1 + RPB2 + TEF1. In addition, divergence time, as an additional criterion, was estimated by the molecular clock analyses with 5-gene loci (5.8S, nLSU, RPB1, RPB2, and TEF1).

## Materials and methods

### Morphological studies

The specimens used in this study are deposited at the herbarium of the Institute of Microbiology, Beijing Forestry University, China (BJFC). Morphological studies and abbreviations of this study followed the study of Sun et al. (2020) and Ji et al. (2022).

### Molecular studies and phylogenetic analysis

The approaches for DNA extraction and polymerase chain reaction (PCR) used in this study with some modifications followed the study of Cui et al. (2019) and Liu et al. (2021). The primer pairs are ITS5 and ITS4 for ITS regions, LR0R and LR7 for nLSU regions, RPB1-Af and RPB1-Cr for the RPB1 gene, bRPB2-6F and bRPB2-7R for the RPB2 gene, and EF1-983F and EF1-1567R for TEF1 (White et al., 1990; Matheny et al., 2002; Matheny, 2005; Rehner and Buckley, 2005). The PCR products were purified and sequenced at the Beijing Genomics Institute (BGI), China. All newly generated sequences were deposited at GenBank (Table 1).

**TABLE 1:** A list of species, specimens, and GenBank accession number of sequences used for phylogenetic analyses in this study.

Species name	Sample no.	Locality	GenBank accessions				
			ITS	nLSU	RPB1	RPB2	TEF1
*Abortiporus biennis*	Cui 17845	China	ON417149	ON417197	ON424663	ON424750	ON424821
*Abortiporus biennis*	Cui 16986	China	ON417150	ON417198	ON424664	ON424751	ON424822
*Adustoporia sinuosa*	Cui 16252	China	OM039269	OM039169	OM037741	OM037767	OM037791
*Adustoporia sinuosa*	Cui 16484	China	MW377252	MW377333	MW337154	ON424753	MW337083
*Agaricostilbum hyphaenes*	AFTOL 675	USA	AY789077	AY634278	AY788845	AY780933	AY879114
*Agaricus campestris*	LAPAG 370	China	KM657927	KR006607	–	KT951556	KR006636
*Amylocorticium cebennense*	CFMR HHB 2808	USA	GU187505	GU187561	GU187439	GU187770	GU187675
*Amyloporia subxantha*	Cui 17175	China	OM039272	OM039172	OM037744	OM037770	OM037794
*Amyloporia xantha*	Cui 11544	China	KR605817	KR605756	ON424665	KR610836	KR610746
*Antrodia serpens*	Dai 7465	Luxemburg	KR605813	KR605752	ON424666	KR610832	KR610742
*Antrodia subserpens*	Cui 16285	China	ON417152	ON417201	ON424669	ON424755	ON424824
*Antrodiella stipitata*	FD 136	USA	KP135314	KP135197	KP134886	–	–
*Aroramyces gelatinosporus*	H 4010	Unknown	–	DQ218524	–	DQ218941	DQ219118
*Athelia arachnoidea*	CBS 418.72	Netherlands	GU187504	GU187557	GU187436	GU187769	GU187672
*Athelia epiphylla*	CFMR FP 100564	USA	GU187501	GU187558	GU187440	GU187771	GU187676
*Aurantiporus albidus*	Cui 16664	Australia	ON682353	**ON680805**	**ON688458**	**ON688479**	**ON688500**
*Aurantiporus albidus*	Cui 16665	Australia	ON682354	**ON680806**	**ON688459**	**ON688480**	**ON688501**
*Bjerkandera adusta*	Cui 16670	Australia	ON682355	**ON680807**	**ON688460**	**ON688481**	**ON688502**
*Bjerkandera adusta*	Cui 16682	Australia	ON682356	**ON680808**	**ON688461**	**ON688482**	**ON688503**
*Boletus edulis*	HMJAU 4637	China	JN563894	KF112455	KF112586	KF112704	KF112202
*Bondarzewia montana*	AFTOL 452	Canada	DQ200923	DQ234539	DQ256049	AY218474	DQ059044
*Bondarzewia* sp.	Yu 56	China	KT693203	KT693205	KX066158	KX066165	KX066148
*Brevicellicium olivascens*	KHL 8571	Sweden	HE963792	HE963793	–	–	–
*Bulbillomyces farinosus*	FP 100488 T	USA	KY948802	DQ681201	KY948929	–	–
*Cabalodontia delicate*	MCW 564/17	Brazil	MT849295	MT849295	MT833947	–	MT833934
*Cabalodontia delicate*	MCW 693/19	Brazil	MT849297	MT849297	MT833948	–	MT833936
*Callistosporium graminicolor*	AFTOL ID 978	USA	DQ484065	AY745702	GU187493	KJ424369	GU187761
*Calocera cornea*	AFTOL 438	USA	AY789083	AY701526	AY857980	AY536286	AY881019
*Ceriporiopsis gilvescens*	Chen 3340	China	MZ636936	MZ637099	MZ748446	OK136039	MZ913651
*Cerrena* sp.	Cui 16874	Puerto Rico	ON682357	**ON680809**	**ON688462**	**ON688483**	**ON688504**
*Cerrena unicolor*	He 6082	China	OM100740	OM083972	ON424672	ON424756	ON424825
*Cerrena zonata*	Cui 16578	Australia	ON417153	ON417203	ON424673	ON424757	ON424826
*Cerrena zonata*	Cui 18502	China	ON417154	ON417204	ON424674	ON424758	ON424827
*Chondrogaster pachysporus*	OSC 49298	Unknown	–	DQ218538	–	DQ218958	DQ219136
*Climacocystis borealis*	Dai 4014	China	KJ566627	KJ566637	**ON688463**	–	KJ566644
*Climacocystis borealis*	FD 31	USA	KP135308	KP135210	KP134882	KP134895	–
*Climacocystis montana*	Cui 9607	China	KJ566629	KJ566639	**ON688464**	**ON688484**	KJ566646
*Climacocystis montana*	Cui 17502	China	MW377276	**MW377356**	**ON688465**	–	–
*Climacocystis montana*	Cui 17122	China	ON682359	**ON680811**	**ON688466**	**ON688485**	**ON688505**
*Climacocystis montana*	Cui 17123	China	ON682360	**ON680812**	**ON688467**	**ON688486**	**ON688506**
*Climacocystis montana*	Cui 17124	China	ON682361	**ON680813**	–	**ON688487**	**ON688507**
*Climacocystis montana*	Dai 23003	China	ON682358	**ON680810**	–	–	–
*Craterocolla cerasi*	TUB 020203	Germany	KF061265	–	–	KF061300	–
*Crustoderma dryinum*	FP 105487	USA	KC585320	KC585145	–	–	–
*Crustoderma dryinum*	HHB 7517	USA	KC585322	KC585147	–	–	–
*Cryptococcus humicola*	AFTOL 1552	USA	DQ645516	DQ645514	DQ645518	DQ645517	DQ645519
*Cymatoderm aelegans*	Dai 17511	China	ON417155	ON417205	–	–	–
*Cymatoderma* sp.	OMC 1427	USA	KY948826	KY948872	KY948971	–	–
*Dacryobolus gracilis*	Dai 14943	China	MH048972	MH048985	–	–	–
*Dacryobolus gracilis*	He 5995	China	ON417156	ON417206	–	ON424760	ON424831
*Dacryobolus karstenii*	Miettinen 18685	USA	KY948743	KY948900	KY948955	–	–
*Dacryobolus montanus*	He 6314	China	ON417157	ON417207	–	ON424761	ON424832
*Dacryobolus sudans*	FP 101996	USA	KC585332	KC585157	–	–	–
*Dacryopinax spathularia*	AFTOL 454	USA	AY854070	AY701525	AY857981	AY786054	AY881020
*Daedalea quercina*	Dai 12152	Czech Republic	KP171207	KP171229	ON424675	KR610809	KR610717
*Daedalea quercina*	Dai 12659	Finland	KP171208	KP171230	ON424676	KR610810	KR610719
*Dictyophora duplicate*	OSC 38819	Unknown	–	DQ218481	–	DQ219087	DQ219265
*Diplomitoporus crustulinus*	Cui 17394	China	MW377287	**MW377366**	**MW337181**	**MW337050**	**MW337114**
*Diplomitoporus crustulinus*	Cui 17475	China	MW377288	**MW377367**	**MW337182**	–	**MW337115**
*Diplomitoporus crustulinus*	Cui 17690	China	MW377289	**MW377368**	**MW337183**	–	**MW337116**
*Diplomitoporus flavescens*	Cui 17457	China	MW377291	**MW377370**	**MW337184**	**MW337052**	**MW337118**
*Diplomitoporus flavescens*	Dai 21020	Belarus	MW377292	**MW377371**	**MW337185**	**MW337053**	**MW337119**
*Diplomitoporus flavescens*	Cui 17419	China	MW377290	**MW377369**	**ON688468**	**MW337051**	**MW337117**
*Diplomitoporus flavescens*	Cui 18420	China	ON682362	**ON680814**	**ON688469**	**ON688488**	**ON688510**
*Diplomitoporus flavescens*	Dai 23640	China	ON682363	**ON680815**	**ON688470**	**ON688489**	**ON688511**
*Echinodontium tinctorium*	AFTOL 455	USA	AY854088	AF393056	AY864882	AY218482	AY885157
*Efibula tropica*	Wei 18-149	China	MZ636967	MZ637129	MZ748419	OK136079	MZ913681
*Efibula yunnanensis*	Wu 880515-1	China	MZ636977	GQ470672	MZ748420	OK136080	MZ913682
*Fibroporia ceracea*	Cui 16299	China	MW377293	MW377372	MW337186	MW337054	MW337120
*Fibroporia ceracea*	Cui 16300	China	MW377294	MW377373	MW337187	MW337055	MW337121
*Fibroporia gossypium*	Cui 9472	China	KU550474	KU550494	ON424677	KU550550	KU550567
*Fibroporia radiculosa*	Cui 16485	Vietnam	OM039278	OM039178	OM037751	OM037776	OM037800
*Fibroporia radiculosa*	Cui 11404	China	KP145011	KR605760	ON424679	KR610840	KR610751
*Fomitiporia hartigii*	MUCL 53551	Belgium	JX093789	JX093833	–	JX093877	JX093746
*Fomitiporia mediterranea*	AFTOL 688	USA	AY854080	AY684157	AY864870	AY803748	AY885149
*Fomitopsis betulina*	Cui 17121	China	OL621853	OL621242	ON424683	OL588969	OL588982
*Fomitopsis eucalypticola*	Cui 16594	Australia	MK852560	MK860110	ON424685	MK900476	MK900483
*Fragiliporia fragilis*	Dai 13080	China	KJ734260	KJ734264	–	KJ790248	KJ790245
*Fragiliporia fragilis*	Dai 13559	China	KJ734261	KJ734265	–	KJ790249	KJ790246
*Fragiliporia fragilis*	Dai 13561	China	KJ734262	KJ734266	–	KJ790250	KJ790247
*Fragiliporia fragilis*	Yuan 5516	China	KJ734263	KJ734267	–	–	–
*Geastrum recolligens*	OSC 41996	Unknown	–	DQ218486	–	DQ219052	DQ219230
*Gelatoporia subvermispora*	Cui 17120	China	ON417159	ON417209	ON424694	ON424772	ON424835
*Gelatoporia subvermispora*	Dai 22847	China	ON417160	ON417210	ON424695	ON424773	ON424836
*Gloeophyllum sepiarium*	Wilcox 3BB	USA	HM536091	HM536061	–	HM536109	HM536110
*Gloeophyllum striatum*	ARIZAN 027866	USA	HM536092	HM536063	–	HM640259	HM536111
*Gloeoporellus merulinus*	Dai 18734	Australia	MW377298	**MW377377**	**MW337191**	**MW337059**	**MW337125**
*Gloeoporellus merulinus*	Dai 18735	Australia	MW377299	**MW377378**	**MW337192**	**ON688490**	**MW337126**
*Gloeoporellus merulinus*	Dai 18782	Australia	MW377300	**MW377379**	**MW337193**	**ON688491**	**MW337127**
*Gloeoporellus merulinus*	Cui 16629	Australia	ON682364	**ON680816**	**ON688471**	**ON688492**	**ON688512**
*Gloeoporellus merulinus*	Cui 16650	Australia	ON682365	**ON680817**	**ON688472**	**ON688493**	**ON688513**
*Gloeoporus dichrous*	Cui 16931	China	ON682366	**ON680818**	**ON688473**	**ON688494**	**ON688514**
*Gloeoporus orientalis*	Wei 16-485	China	MZ636980	MZ637141	MZ748443	OK136095	MZ913709
*Grifola frondosa*	AFTOL 701	Unknown	AY854084	AY629318	AY864876	–	AY885153
*Grifola frondosa*	Dai 19172	Canada	ON417161	ON417211	ON424696	ON424774	ON424837
*Grifola frondosa*	Dai 19175	Canada	ON417162	ON417212	ON424697	ON424775	ON424838
*Gymnopilus picreus*	ZRL 2015011	China	LT716066	KY418882	KY418980	KY419027	KY419077
*Heterobasidion annosum*	Dai 20962	Belarus	ON417163	ON417213	ON424698	ON424776	ON529284
*Hydnum repandum*	BB 07.341	Unknown	–	KF294643	–	KF294720	JX192980
*Hymenochaete rubiginosa*	He 1049	China	JQ716407	JQ279667	–	–	–
*Hyphoderma litschaueri*	FP 101740	USA	KP135295	KP135219	KP134868	KP134965	–
*Hyphoderma medioburiense*	FD 335	USA	KP135298	KP135220	KP134869	KP134966	–
*Hyphoderma mutatum*	HHB 15479	USA	KP135296	KP135221	KP134870	KP134967	–
*Hyphoderma setigerum*	FD 312	USA	KP135297	KP135222	KP134871	–	–
*Hypochnicium bombycinum*	HHB 12631	USA	KY948801	KY415959	KY948930	–	–
*Hypochnicium geogenium*	He 6804	China	OM039279	**OM039179**	–	**OM037777**	**OM037802**
*Hypochnicium geogenium*	He 6812	China	**OM039280**	**OM039180**	–	**OM037778**	**OM037803**
*Hypochnicium geogenium*	He 6817	China	**OM039281**	**OM039181**	–	**OM037779**	**OM037804**
*Hypochnicium geogenium*	He 6819	China	**OM039282**	**OM039182**	–	**OM037780**	**OM037805**
*Hypochnicium karstenii*	HHB 9373	USA	KY948799	DQ677510	KY948931	–	–
*Hypochnicium punctulatum*	FP 101698	USA	KY948827	KY948860	KY948932	–	–
*Hypochnicium sphaerosporum*	RLG 15138	USA	KY948803	KY948861	KY948940	–	–
*Hypochnicium wakefieldiae*	KJM 271	USA	KY948828	DQ677512	KY948933	–	–
*Irpex flavus*	Wu 0705-1	China	MZ636988	MZ637149	MZ748432	OK136087	MZ913683
*Irpex* sp.	Wu 910807-35	China	MZ636994	GQ470627	MZ748433	OK136088	MZ913684
*Ischnoderma benzoinum*	Cui 17058	China	ON417164	ON417214	ON424699	ON424777	ON424839
*Ischnoderma benzoinum*	Cui 17700	China	ON417165	ON417215	ON424700	ON424778	ON424840
*Jaapia argillacea*	CBS 252.74	Netherlands	GU187524	GU187581	GU187463	GU187788	GU187711
*Lactarius deceptivus*	AFTOL ID 682	USA	AY854089	AY631899	AY864883	AY803749	AY885158
*Laetiporus montanus*	Cui 10015	China	KF951273	KF951311	ON424701	KT894791	KX354618
*Laetiporus montanus*	Cui 10011	China	KF951274	KF951315	MG867670	KT894790	KX354617
*Laetiporus sulphureus*	Cui 12389	China	KR187106	KX354487	ON424702	KX354653	KX354608
*Laetiporus sulphureus*	Cui 12388	China	KR187105	KX354486	MG867671	KX354652	KX354607
*Laricifomes officinalis*	JV 0309/49-J	USA	KR605821	KR605764	–	KR610846	KR610757
*Laricifomes officinalis*	JV 9010/14	Slovak Republic	KR605822	KR605765	–	KR610847	KR610758
*Lentoporia carbonica*	Zabel 40 GLN	USA	KC585243	KC585065	KY949013	–	–
*Lentoporia carbonica*	DAOM F 8281	Cabada	KC585239	KC585061	–	–	–
*Lepiota cristata*	ZRL 20151133	China	LT716026	KY418841	KY418963	KY418992	KY419048
*Leptoporus mollis*	TJV-93-174-T	USA	KY948795	EU402510	KY948957	OK136102	MZ913694
*Leptoporus mollis*	RLG 7163	USA	KY948794	MZ637155	KY948956	OK136101	MZ913693
*Leptosporomyces raunkiaeri*	CFMR HHB 7628	USA	GU187528	GU187588	GU187471	GU187791	GU187719
*Luteoporia albomarginata*	GC 1702-1	China	LC379003	LC379155	LC379160	LC387358	LC387377
*Luteoporia lutea*	GC 1409-1	China	MZ636998	MZ637158	MZ748467	OK136050	MZ913656
*Macrohyporia dictyopora*	Dai 18878	Australia	OK036736	OK036735	–	–	OK076964
*Meripilus giganteus*	FP 135344	United Kingdom	KP135307	KP135228	KP134873	–	–
*Metuloidea reniforme*	MCW 523/17	Brazil	MT849302	MT849302	MT833949	–	–
*Metuloidea reniforme*	MCW 542/17	Brazil	MT849303	MT849303	MT833950	–	MT833940
*Multiclavula mucida*	AFTOL 1130	USA	DQ521417	AY885163	–	–	–
*Neurospora crassa*	OR 74A	India	HQ271348	AF286411	XM959004	AF107789	XM959775
*Obba rivulosa*	Cui 16477	Vietnam	**ON682367**	**ON680819**	**ON688474**	**ON688495**	**ON688515**
*Obba rivulosa*	Cui 16483	Vietnam	ON417171	ON417221	ON424711	ON424787	ON424849
*Obba rivulosa*	Cui 16482	Vietnam	ON417172	ON417222	ON424712	ON424788	ON424850
*Panus fragilis*	HHB 11042	USA	KP135328	KP135233	KP134877	–	–
*Perenniporia yinggelingensis*	Cui 13627	China	MH427961	MH427968	MH427989	MH427993	MH427997
*Perenniporia yinggelingensis*	Cui 13631	China	MH427962	MH427969	MH427990	MH427994	MH427998
*Phaeolus schweinitzii*	Dai 8025	China	KX354457	KX354511	–	DQ408119	KX354686
*Phaeophlebiopsis caribbeana*	HHB 6990	USA	KP135415	KP135243	KP134810	KP134931	MZ913643
*Phaeophlebiopsis ravenelii*	FCUG 2126	Spain	MZ637015	GQ470675	MZ748361	OK135993	MZ913634
*Phallus costatus*	MB 02040	Unknown	–	DQ218513	–	DQ219104	DQ219279
*Phanerochaete alnea*	FP 151125	USA	KP135177	MZ637181	MZ748385	OK136014	MZ913641
*Phanerochaete canolutea*	Wu 9211-105	China	MZ422795	GQ470641	MZ748387	OK136018	MZ913640
*Phanerochaetella angustocystidiata*	Wu 9606-39	China	MZ637020	GQ470638	MZ748422	OK136082	MZ913687
*Phanerochaetella leptoderma*	Chen 1362	China	MZ637025	GQ470646	MZ748423	OK136083	MZ913689
*Phlebia nantahaliensis*	HHB 2816	USA	KY948777	KY948852	KY948920	OK136063	MZ913701
*Phlebia tomentopileata*	GC 1602-67	China	MZ637040	MZ637244	MZ748457	OK136064	MZ913702
*Phlebiopsis gigantea*	FCUG 1417	Norway	MZ637051	AF141634	MZ748370	OK135996	MZ913623
*Phlebiopsis odontoidea*	GC 1708-181	China	MZ637054	MZ637255	MZ748371	OK135997	MZ913624
*Physisporinus longicystidius*	Cui 16630	Australia	ON417177	ON417227	ON424717	ON424795	ON424856
*Physisporinus longicystidius*	Cui 16725	Australia	ON417178	ON417228	ON424718	ON424796	ON424857
*Picipes badius*	Cui 10853	China	KU189780	KU189811	KU189894	KX900300	KU189929
*Picipes badius*	Cui 11136	China	KU189781	KU189812	KU189895	KU189990	KU189930
*Podoscypha venustula*	Cui 16923	Puerto Rico	ON417181	ON417231	ON424722	ON424799	ON424860
*Podoserpula ailaoshanensis*	ZJL 2015015	China	KU324484	KU324487	–	–	KU324494
*Polyporus squamosus*	Cui 10595	China	KU189778	KU189809	KU189892	KU189988	KU189925
*Polyporus varius*	Cui 12249	China	KU507581	KU507583	KU507589	KU507592	KU507591
*Porodaedalea chinensis*	Cui 10252	China	KX673606	MH152358	–	MH101479	MG585301
*Pseudofibroporia citrinella*	He 20120721	China	KU550477	KU550500	–	KU550555	KU550574
*Pseudofibroporia citrinella*	Yuan 6181	China	KU550478	KU550501	–	KU550556	KU550575
*Pycnoporellus fulgens*	Cui 16463	Vietnam	MW377318	MW377396	ON424725	ON424805	ON424867
*Pycnoporellus fulgens*	Cui 10033	China	KX354458	KX354512	ON424726	KX354684	KX354687
*Pyrenogaster pityophilus*	OSC 59743	Unknown	–	DQ218519	–	DQ219057	DQ219232
*Radulodon casearius*	HHB 9567	USA	KY948752	KY948871	KY948943	–	–
*Resinoporia sordida*	Dai 23393	China	**ON682368**	**ON680820**	**ON688475**	**ON688496**	**ON688516**
*Resinoporia sordida*	Cui 16469	Vietnam	ON417186	ON417237	ON424730	ON424806	ON424870
*Rhizochaete chinensis*	Wu 0910-45	China	LC387335	MF110294	LC387348	LC387370	LC270925
*Rhizochaete sulphurina*	HHB 5604	USA	KY273031	GU187610	MZ748363	OK135991	MZ913707
*Rhizopus stolonifer*	CBS 609.82	Japan	AB113023	DQ273817	–	–	AB512268
*Rhodofomes roseus*	Cui 17046	China	ON417187	ON417238	ON424731	ON424807	ON424871
*Rhodofomes roseus*	Cui 17081	China	ON417188	ON417239	ON424732	ON424808	ON424872
*Rhodofomitopsis pseudofeei*	Cui 16794	Australia	MK461952	MK461956	ON424735	MK463984	MK463986
*Rhodofomitopsis pseudofeei*	Cui 16762	Australia	MK461951	MK461955	**ON688476**	MK463983	MK463985
*Rhodonia obliqua*	Dai 23436	China	ON417191	ON417242	ON424738	–	ON424876
*Rhodonia placenta*	Wei 1406	China	KF699129	KT893750	ON424739	KT893746	KT893748
*Rickiopora latemarginata*	RP 56	Brazil	KU521768	KU521768	–	–	–
*Rickiopora latemarginata*	RP 58	Brazil	KU521769	KU521769	–	KU521773	KU521771
*Rickiopora latemarginata*	RP 110	Brazil	KU521770	KU521770	–	KU521774	KU521772
*Rigidoporus corticola*	ZRL 20151459	China	LT716075	KY418899	KY419038	KY419087	KY418954
*Rigidoporus ginkgonis*	Cui 5555	China	KT203295	KT203316	–	–	–
*Rigidoporus* sp.	Cui 16852	Puerto Rico	ON417179	ON417229	ON424719	ON424797	ON424858
*Rigidoporus* sp.	Cui 16859	Puerto Rico	ON417180	ON417230	ON424720	ON424798	ON424859
*Rigidoporus undatus*	Miettinen 13591	Finland	KY948731	KY948870	KY948945	–	–
*Russula emeticicolor*	FH 12253	Germany	KT934011	KT933872	KT957382	KT933943	–
*Ryvardenia campyla*	Cui 16674	Australia	MW377323	MW377400	MW337203	MW337076	MW337143
*Ryvardenia cretacea*	Cui 16731	Australia	MW377324	MW377401	MW337204	MW337077	MW337144
*Ryvardenia cretacea*	Cui 16732	Australia	MW377325	MW377402	MW337205	MW337078	MW337145
*Sarcoporia polyspora*	Cui 16977	China	MW377326	MW377403	MW337206	MW337079	MW337146
*Sarcoporia polyspora*	Cui 16995	China	OM039299	OM039199	OM037761	ON424811	OM037817
*Sarcoporia polyspora*	Cui 17165	China	ON417192	ON417244	ON424740	ON424812	ON424878
*Schizosaccharomyces pombe*	972 h	France	Z19578	Z19136	NM001021568	NM001018498	NM001021161
*Scopuloides allantoidea*	Wei 16-060	China	MZ637081	MZ637279	MZ748463	OK136047	MZ913664
*Scopuloides rimosa*	HHB 15484	USA	KP135352	KP135281	KP134851	KP134902	MZ913665
*Scopuloides rimosa*	Wu 1507-117	China	MZ637087	MZ637284	MZ748464	OK136048	MZ913666
*Serpula himantioides*	MUCL 30528	Belgium	GU187545	GU187600	GU187480	GU187808	GU187748
*Skeletocutis coprosmae*	Cui 16623	Australia	ON417193	ON417245	ON424741	ON424813	ON424879
*Skeletocutis nivea*	Cui 16752	Australia	**ON682369**	**ON680821**	**ON688477**	**ON688497**	**ON688517**
*Skeletocutis yuchengii*	FBCC 1132	China	KY953045	KY953045	KY953143	–	KY953109
*Skeletocutis yunnanensis*	Dai 15709	China	KU950434	KU950436	MW526263	–	MW427605
*Sparassis crispa*	AFTOL ID 703	Unknown	DQ250597	AY629321	–	DQ408122	DQ056289
*Sparassis crispa*	MBUH DORISL	Germany	AY218442	AY218404	–	–	–
*Sparassis radicata*	SS 29	Unknown	AY218446	AY218408	–	DQ270672	–
*Sparassis radicata*	TENN 52558	USA	AY218450	AY218411	–	AY218547	–
*Sparassis radicata*	OKM 4756	USA	KC987580	KF053407	KY949023	–	–
*Steccherinum larssonii*	MCW 593/17	Brazil	MT849306	MT849306	MT833956	–	MT833941
*Steccherinum meridionale*	Cui 16691	Australia	ON417195	ON417247	ON424743	ON424817	ON424882
*Steccherinum* sp.	Cui 16755	Australia	**ON682370**	**ON680822**	**ON688478**	**ON688498**	**ON688518**
*Stereopsis radicans*	OLR 45395	Belize	–	KC203496	–	KC203502	KC203516
*Stereopsis* sp.	OKHL 15544	Brazil	–	–	–	KC203505	KC203519
*Stereum hirsutum*	FPL 8805	Unknown	–	AF393078	–	–	–
*Stereum hirsutum*	AFTOL ID 492	Unknown	AY854063	–	AY864885	AY218520	AY885159
*Suillus pictus*	AFTOL 717	Unknown	AY854069	AY684154	AY858965	AY786066	AY883429
*Thelephora ganbajun*	ZRL 20151295	China	LT716082	KY418908	KY418987	KY419043	KY419093
*Tomentella* sp.	AFTOL ID 1016	USA	DQ835998	DQ835997	–	DQ835999	–
*Trametes cinnabarina*	Dai 14386	China	KX880629	KX880667	KX880818	KX880854	KX880885
*Trametes sanguinea*	Cui 7091	China	KX880628	KX880666	KX880817	MG867689	KX880884
*Trechispora alnicola*	AFTOL 665	Unknown	DQ411529	AY635768	–	–	DQ059052
*Tremellodendron* sp.	PBM 2324	Unknown	DQ411526	–	–	DQ408132	DQ029196
*Tyromyces chioneus*	FD 4	USA	KP135311	KP135291	KP134891	KP134977	–
*Tyromyces odora*	L 13763	Canada	KY948830	KY948893	KY949046	–	–
*Tyromyces* sp.	Cui 16652	Australia	ON417196	ON417248	ON424749	ON424820	ON424885
*Wolfiporia cocos*	CBS 279.55	USA	MW251869	MW251858	–	MW250264	MW250253
*Wolfiporia hoelen*	Dai 20036	China	MW251877	MW251866	–	MW250272	MW250261
*Wolfiporia hoelen*	Dai 20034	China	MW251879	MW251868	–	**ON688499**	MW250263
*Wolfiporia dilatohypha*	CS 635913	USA	KC585400	KC585234	KY949026	–	–
*Wolfiporia castanopsidis*	Cui 16295	China	–	MW377408	MW337209	MW337080	MW337151
*Wolfiporia castanopsidis*	Cui 16296	China	–	MW377409	MW337210	MW337081	MW337152

Newly generated sequences for this study are shown in bold.

In the phylogenetic analyses, we selected exact and more gene fragments of representative species from previous studies. Other sequences were obtained from GenBank (Table 1). The sequences used in this study were aligned in MAFFT 7 (Katoh and Standley, 2013)^1^ and then manually adjusted in BioEdit (Hall, 1999). Each alignment sequence was spliced with Mesquite (Maddison and Maddison, 2017). The missing sequences and ambiguous nucleotides were coded as “N.”

The phylogenetic analysis methods used in this study followed Shen et al. (2019) and Sun et al. (2022). The sequences of *Heterobasidion annosum* (Fr.) Bref. and *Stereum hirsutum* (Willd.) Pers. were obtained as outgroups for the phylogenetic analyses following Binder et al. (2013) and Justo et al. (2017). The congruences of gene sequence datasets were evaluated with the incongruence length difference (ILD) test (Farris et al., 1994) with PAUP* 4.0b10 (Swofford, 2002), under 1,000 homogeneity replicates. Maximum parsimony analysis was applied to the combined gene dataset and the tree construction procedure was performed in PAUP* version 4.0b10. Clade robustness was assessed using a bootstrap (BT) analysis with 1,000 replicates (Felsenstein, 1985). Descriptive tree statistic tree length (TL), consistency index (CI), retention index (RI), rescaled consistency index (RC), and homoplasy index (HI) were calculated for each Most Parsimonious Tree (MPT) generated.

Maximum likelihood (ML) analysis was performed with RAxML-HPC v. 8.2.3 (Stamatakis, 2014) with 1,000 ML searches under the GTRGAMMA model, and only the maximum likelihood best tree from all searches was kept. Bayesian inference (BI) was performed using MrBayes v. 3.2 (Ronquist and Huelsenbeck, 2003) with four simultaneous independent chains for all datasets, performing five million generations until the split deviation frequency value of <0.01, and sampled every 100th generation. The first 25% of sampled trees were discarded as burn-in, while the remaining ones were used to calculate Bayesian posterior probabilities (BPPs) of the clades.

Phylogenetic trees were inferred from the combined sequences datasets of ITS + nLSU + RPB1 and ITS + nLSU + RPB1 + RPB2 + TEF1. Trees were viewed in FigTree v1.4.4.^2^ Branches that received bootstrap supports for maximum parsimony (MP), maximum likelihood (ML), and Bayesian posterior probabilities (BPP) greater than or equal to 75% (MP and ML) and 0.95 (BPP) were considered as significantly supported, respectively.

### Divergence time estimation

Three fossil calibrations, *Archaeomarasmius leggetti* Hibbett, D. Grimaldi and Donoghue, *Quatsinoporites cranhamii* S. Y. Sm., Currah and Stockey, and *Paleopyrenomycites devonicus* Taylor, Hass, Kerp, M. Krings and Hanlin, were used in the divergence time estimating. *Archaeomarasmius leggetti* was used as the representative of the minimum age of Agaricales at 90–94 Mya (Hibbett et al., 1997); *Q. cranhamii* was the representative of the minimum age of Hymenochaetales at 113 Mya (Smith et al., 2004); *P. devonicus* was used as the representative of the minimum age between Basidiomycota and Ascomycota at 400 Mya (Taylor et al., 2005; Berbee and Taylor, 2010). Divergence time is estimated with the BEAST v1.8.0 software package (Drummond et al., 2012) with 5.8S, nLSU, RPB1, RPB2, and TEF1 sequences representing main lineages in Basidiomycota (Table 1).

All the DNA sequences of 5.8S, nLSU, RPB1, RPB2, and TEF1 were aligned in MAFFT 7 (Katoh and Standley, 2013) and manually adjusted in BioEdit (Hall, 1999). ModelTest 3.7 was used to estimate the rate of evolutionary changes at nucleic acids with the GTR substitution model (Posada and Crandall, 1998). BEATUti v2 was used to generate the BEAST XML input file. A log-normal distribution is employed for molecular clock analysis (Drummond and Rambaut, 2007). The clock model was set to an uncorrelated lognormal relaxed clock (Drummond et al., 2006; Lepage et al., 2007). A Yule speciation model is selected as the prior choice assuming a constant speciation rate per lineage. Gamma prior distribution was used for fossil node calibrations (shape = 1.0, scale = 50.0), and the offset was set at 90.0, 113.0, and 400.0 for Agaricales, Hymenochaetales, and Basidiomycota, respectively (Sánchez-Ramírez et al., 2014). All the ucld. mean parameters for different genes were set to gamma prior distribution, shape = 1.0, scale = 0.001, and offset = 0.0 (Sánchez-Ramírez et al., 2014). Overall, four independent Markov chain Monte Carlo (MCMC) chains of 100 million generations were conducted and saving trees every 5,000th generation. The log file is analyzed in Tracer v1.6 to confirm that the estimated effective sample size (ESS) is ≥200^3^. A maximum clade credibility (MCC) tree is summarized in TreeAnnotator, removing the first 10% of the sampled trees as burn-in and setting a posterior probability limit of 0.80, and viewed in FigTree v1.4.4.

## Results

### Phylogeny

**FIGURE 1:**
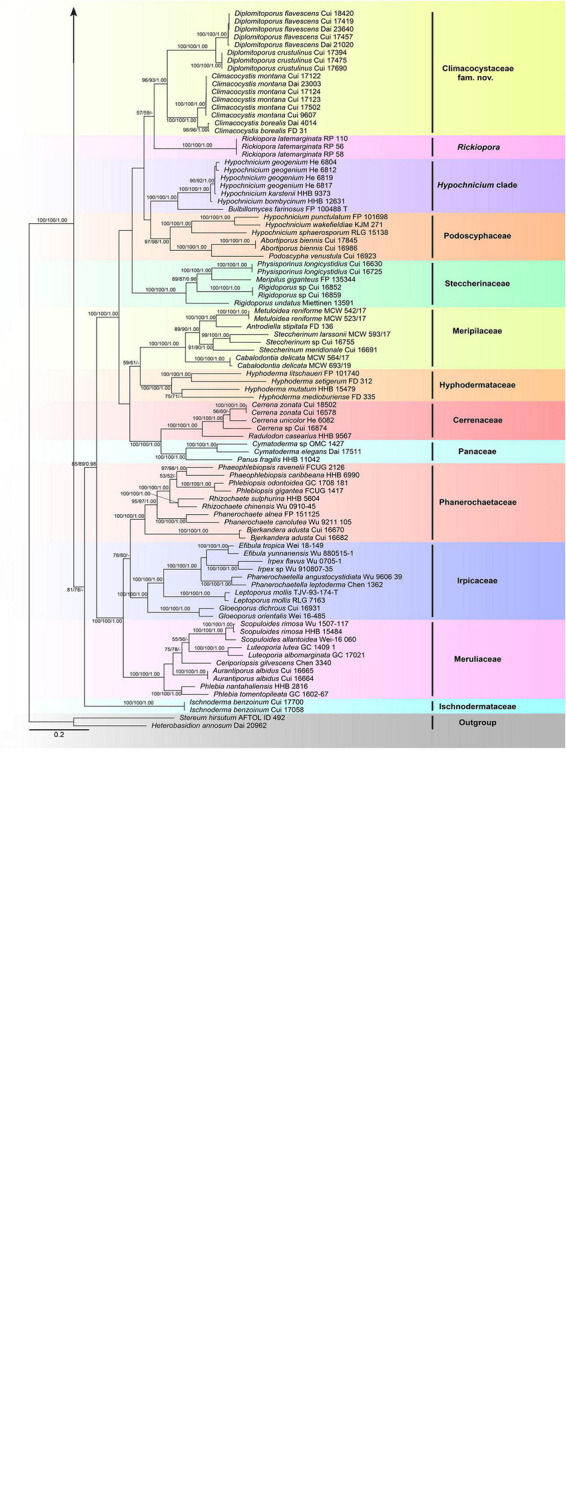
Maximum likelihood tree illustrating the phylogeny of the Polyporales based on the combined sequence dataset of ITS + nLSU + RPB1. Branches are labeled with parsimony bootstrap proportions higher than 50%, maximum likelihood bootstrap higher than 50% and Bayesian posterior probabilities more than 0.90, respectively.

The combined 3-gene (ITS + nLSU + RPB1) sequence dataset had an aligned length of 3,289 characters, including gaps (628 characters for ITS, 1,333 characters for nLSU, and 1,328 characters for RPB1), of which 1,415 characters were constant, 130 were variable and parsimony-uninformative, and 1,744 were parsimony-informative. MP analysis yielded 54 equally parsimonious trees (*TL* = 25,472, *CI* = 0.154, *RI* = 0.653, *RC* = 0.101, *HI* = 0.846). The best-fit evolutionary models applied in the Bayesian analyses were selected by MrModeltest2 v. 2.3 for each region of the three genes, and the model for ITS, nLSU, and RPB1 was GTR + I + G with an equal frequency of nucleotides. This model was applied in the Bayesian analyses for the combined dataset. ML analysis resulted in a similar topology as MP and Bayesian analyses, and only the ML topology is shown in Figure 1.

**FIGURE 2:**
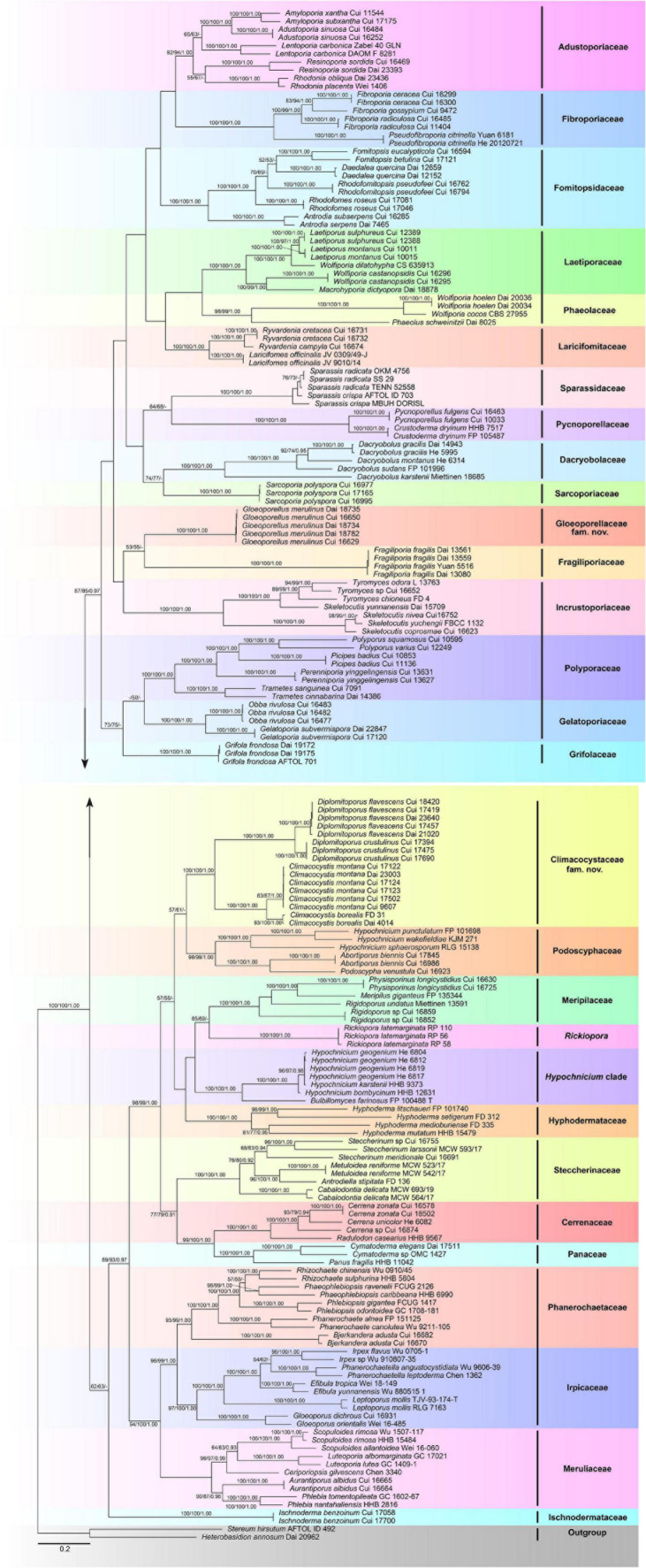
Maximum likelihood tree illustrating the phylogeny of the Polyporales based on the combined sequence dataset of ITS + nLSU + RPB1 + RPB2 + TEF1. Branches are labeled with parsimony bootstrap proportions higher than 50%, maximum likelihood bootstrap higher than 50%, and Bayesian posterior probabilities more than 0.90, respectively.

The combined 5-gene (ITS + nLSU + RPB1 + RPB2 + TEF1) sequence dataset had an aligned length of 4,849 characters, including gaps (628 characters for ITS, 1,333 characters for nLSU, 1,328 characters for RPB1, 1,020 characters for RPB2, and 540 characters for TEF1), of which 1,980 characters were constant, 177 were variable and parsimony-uninformative, and 2,692 were parsimony-informative. MP analysis yielded 80 equally parsimonious trees (*TL* = 41,080, *CI* = 0.149, *RI* = 0.634, *RC* = 0.094, *HI* = 0.851). The best-fit evolutionary models applied in the Bayesian analyses were selected by MrModeltest2 v. 2.3 for each region of the five genes, and the model for ITS, LSU, RPB1, RPB2, and TEF1 was GTR + I + G with an equal frequency of nucleotides. This model was applied in the Bayesian analyses for the combined dataset. ML analysis resulted in a similar topology as MP and Bayesian analyses, and only the ML topology is shown in Figure 2.

The phylogenetic trees inferred from ITS + nLSU + RPB1 and ITS + nLSU + RPB1 + RPB2 + TEF1 gene sequences were obtained from 185 fungal samples representing 113 taxa of Polyporales and two taxa of Russulales (Figures 1, 2). A total of 810 sequences derived from five gene loci (ITS, nLSU, RPB1, RPB2, and TEF1) were used to reconstruct the phylogenetic trees, of which 153 were newly generated. Phylogenetic analyses showed that 27 clades within Polyporales are assigned family names, including two new families, viz., Climacocystaceae fam. nov. and Gloeoporellaceae fam. nov., established for the climacocystis lineage (*Climacocystis* and *Diplomitoporus*) and *Gloeoporellus merulinus* (Berk.) Zmitr. (= *Tyromyces merulinus*) (Figures 1, 2).

### Divergence time estimation

**FIGURE 3:**
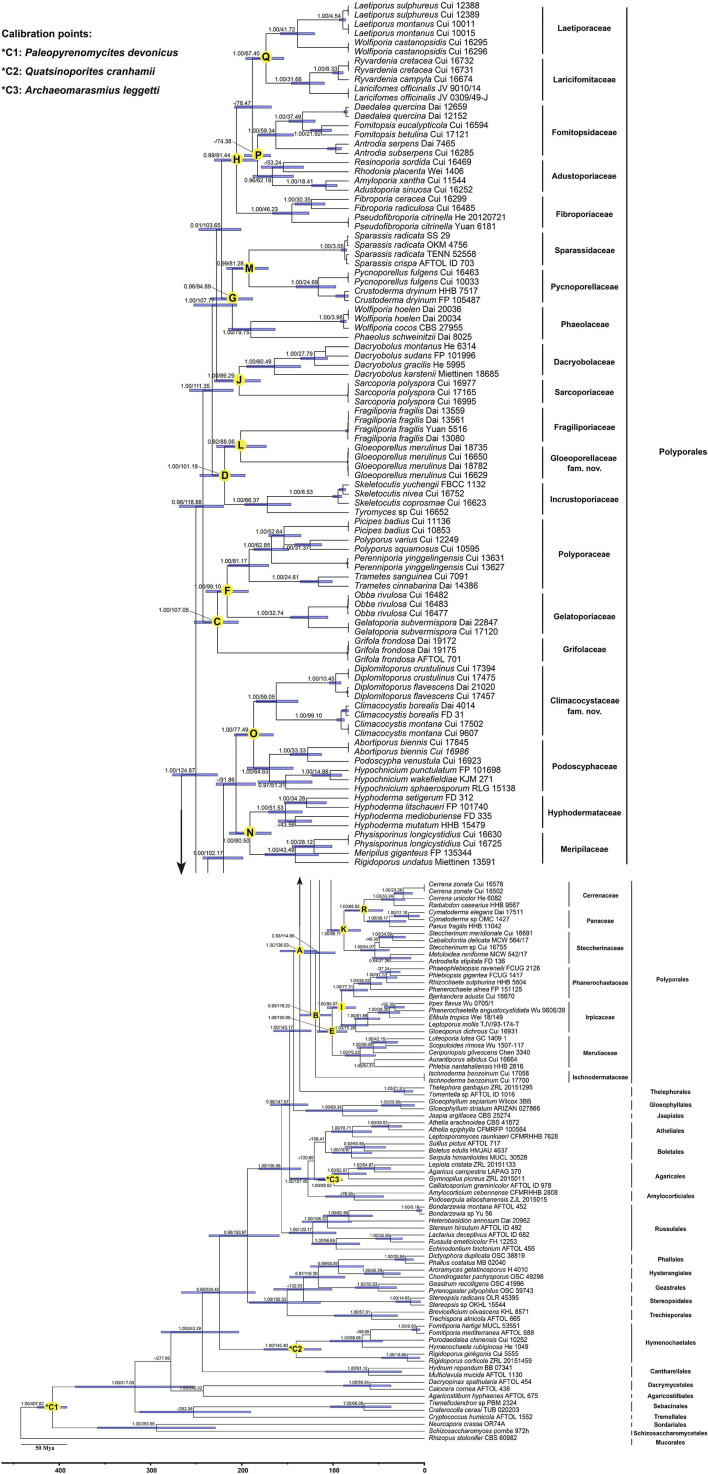
Divergence time estimation of families within Polyporales from Bayesian evolutionary analysis sampling tree based on the combined sequence dataset of 5.8S + nLSU + RPB1 + RPB2 + TEF1. Posterior probabilities not less than 0.80 and the mean ages of each node are annotated. The 90% highest posterior densities of divergence time estimation are marked by horizontal bars.

The combined dataset (5.8S, nLSU, RPB1, RPB2, and TEF1) for the molecular clock analysis includes 174 fungal samples representing 132 taxa, of which 123 fungal samples represent 87 taxa belonging to Polyporales. The MCMC tree (Figure 3) shows that the most recent ancestor of Polyporales evolved during the early Cretaceous, approximately 136.53 Mya with a 95% highest posterior density (HPD) of 118.08–158.06 Mya. The youngest families of Polyporales are Cerrenaceae Miettinen, Justo and Hibbett and Panacea Miettinen, Justo and Hibbett, occurring in a mean stem age of 66.02 Mya with a 95% HPD of 45.28–86.67 Mya, the oldest family of Polyporales is Ischnodermataceae Jülich, occurring in a mean stem age of 119.22 with a 95% HPD of 102.03–136.08 Mya, and the average divergence time of the families in Polyporales is 86.34 Mya. The estimated divergence times for families of Polyporales are summarized in Table 2.

**TABLE 2:** The divergence times of estimated taxa in Polyporales.

Node	Mean of stem age (Mya)	95% HPD (Mya)
A: Polyporales/Thelephorales	136.53	118.08–158.06
B: Ischnodermataceae	119.22	102.03–136.08
C: Grifolaceae	107.05	89.96–126.02
D: Incrustoporiaceae	101.19	84.47–121.39
E: Meruliaceae	100.98	84.76–117.47
F: Gelatoporiaceae/Polyporaceae	99.1	81.93–116.47
G: Phaeolaceae	94.89	78.85–112.34
H: Fibroporiaceae	91.44	73.90–109.55
I: Irpicaceae/Phanerochaetaceae	90.57	75.39–106.39
J: Dacryobolaceae/Sarcoporiaceae	89.29	71.96–109.52
K: Steccherinaceae	88.11	69.75–106.60
L: Fragiliporiaceae/Gloeoporellaceae	88.06	67.15–107.76
M: Pycnoporellaceae/Sparassidaceae	81.28	65.62–99.46
N: Hyphodermataceae/Meripilaceae	80.5	63.16–97.26
O: Climacocystaceae/Podoscyphaceae	77.49	61.45–93.16
P: Adustoporiaceae/Fomitopsidaceae	74.38	58.72–90.04
Q: Laetiporaceae/Laricifomitaceae	67.4	52.85–83.94
R: Cerrenaceae/Panaceae	66.02	45.28–86.67

### Taxonomy

Climacocystaceae B.K. Cui, Shun Liu & Y.C. Dai, fam. nov.

MycoBank: MB 840367

*Type genus*: *Climacocystis*.

*Diagnosis*: Basidiomata annual, pileate, resupinate to effused-reflexed, soft corky and watery when fresh, brittle, corky to hard corky when dry. Hymenophores poroid. Hyphal system monomitic, dimitic to trimitic; generative hyphae with clamp connections, skeletal hyphae IKI–, CB–. Cystidia present or absent, cystidioles occasionally present. Basidiospores broadly ellipsoid to globose, colorless, thin- to slightly thick-walled, smooth, IKI–, CB–. Causing a white rot.

*Genera*: *Climacocystis*, *Diplomitoporus*.

*Climacocystis* Kotl. and Pouzar, Ceská Mykologie 12 (2): 95, 1958.

MycoBank: MB 17325

*Type species*: *Climacocystis borealis* (Fr.) Kotl. and Pouzar.

*Diagnosis*: Basidiomata annual, pileate, sessile to laterally substipitate, usually imbricate, soft and watery when fresh, corky to hard corky and light in weight when dry. Pileus applanate, fan-shaped to dimidiate. Pileal surface white to cream, tomentose to hirsute when fresh, becoming cream, yellowish-brown to orange-brown, glabrous or tufted with short stiff hairs when dry, often radially furrowed, azonate; margin acute. Pore surface white to cream when fresh, becoming cream, clay-buff to orange-brown when dry; pores angular or irregular; dissepiments thin, entire to lacerate. Context white to clay-buff, corky to hard corky. Tubes white, clay-buff to orange-brown, corky to hard corky. Hyphal system monomitic; generative hyphae with clamp connections, IKI–, CB–; tissues unchanged in KOH. Cystidia present, ventricose, colorless, thin- to thick-walled, smooth or apically encrusted. Basidia clavate, colorless, thin-walled. Basidiospores ellipsoid to subcylindrical, colorless, thin-walled, smooth, IKI–, CB–. Causing a white rot.

**FIGURE 4:**
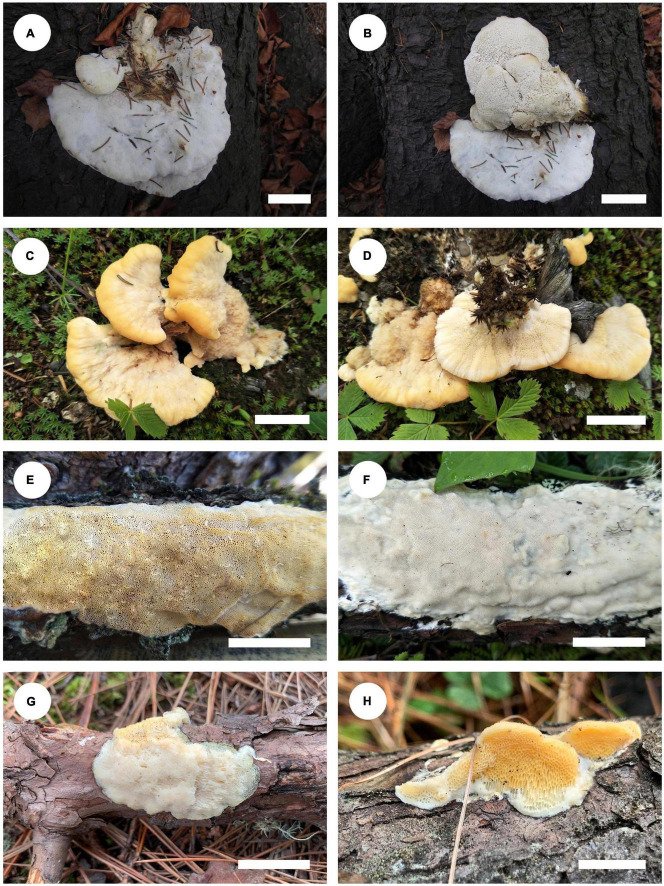
Basidiomata of *Climacocystis* and *Diplomitoporus* species: *C. borealis* (**A,B**: Dai 13028); *C. montana* (**C,D**: Cui 17502); *D. crustulinus* (**E**: Cui 17394; **F**: Cui 17475); *D. flavescens* (**G**: Dai 22798; **H**: Dai 23640). Scale bars: ***A*** = 2 cm; ***B***,***C***,***D*** = 3 cm; ***E***,***F***,***G***,***H*** = 1.5 cm.

*Notes*: *Climacocystis* was established by Kotlába and Pouzar (1958) and typified by *C. borealis*, which is widely distributed in the northern hemisphere (Gilbertson and Ryvarden, 1986; Núñez and Ryvarden, 2001; Dai, 2012). Song et al. (2014) carried out taxonomic and phylogenetic studies on *Climacocystis* in China, and *C. montana* B-KC and JS described high elevations in southwestern China based on morphological and molecular characteristics. Currently, two species are accepted in *Climacocystis*, including *C. borealis* and *C. montana*. Basidiomata of *C. borealis* and *C. montana* are shown in Figure 4.

*Specimens examined*: *Climacocystis borealis*. CHINA. Heilongjiang Province, Yichun, Wuying, Fenglin Nature Reserve, on stump of *Pinus* sp., 8 September 2002, Dai 3703 (BJFC 000443). FINLAND. Helsinki, Vantaa, Tamisto Nature Reserve, on fallen trunk of *Picea* sp., 22 September 2010, Dai 11798 (BJFC 008905); on stump of *Picea* sp., 15 November 2011, Dai 12681 (BJFC 012265). SWITZERLAND. Geneva, on living tree of *Picea* sp., 25 November 2012, Dai 13208 (BJFC 014072). *Climacocystis montana*. CHINA. Xizang Autonomous Region (Tibet), Leiwuqi County, on fallen trunk of *Picea* sp., 22 September 2010, Cui 9603 (BJFC 008541, holotype), Cui 9610 (BJFC 008548), Cui 9612 (BJFC 008550), Cui 9607 (BJFC 008545). Sichuan Province, Jiuzhaigou County, Jiuzhaigou Nature Reserve, on fallen trunk of *Picea* sp., 11 October 2012, Cui 10603 (BJFC 013528). Yunnan Province, Shangri-La County, Pudacuo National Park, on root of *Picea* sp., 17 September 2018, Cui 17122 (BJFC 030422), Cui 17123 (BJFC 030423), Cui 17124 (BJFC 030424), 13 August 2019, Cui 17502 (BJFC 034361); on root of Picea sp., 7 September 2021, Dai 23003 (BJFC 037576).

*Diplomitoporus* Domanski, Acta Societatis Botanicorum Poloniae 39: 191, 1970.

MycoBank: MB 17515

*Type species*: *Diplomitoporus flavescens* (Bres.) Domański.

*Diagnosis*: Basidiomata annual, resupinate to effuse-reflexed, fibrous, soft corky when fresh, brittle to hard corky when dry. Pore surface white, cream to straw-yellow when fresh, becoming cream, ochraceous to dark ochraceous when dry; pores round to angular. Context cream to ochraceous, brittle to corky. Tubes concolorous with the pore surface, brittle to hard corky. Hyphal system dimitic to trimitic; generative hyphae with clamp connections; skeletal hyphae IKI–, CB–. Cystidia absent; cystidioles occasionally present, subclavate to fusoid, colorless, thin-walled, smooth. Basidia clavate, subclavate to subglobose, colorless, thin-walled. Basidiospores allantoid to ellipsoid or globose, colorless, thin- to slightly thick-walled, smooth, IKI–, CB–. Causing a white rot.

*Notes*: *Diplomitoporus* was described by Domanski (1970) with *D. flavescens* as type species. Some species of *Diplomitoporus* have been transferred to other genera based on morphological or molecular evidence (Ghobad-Nejhad and Dai, 2010; Miettinen, 2012). Baltazar et al. (2014) reviewed the species of *Diplomitoporus* from Brazil and reported seven species of this genus in Brazil. In recent years, Ryvarden and co-authors described several *Diplomitoporus* species based on their morphological characteristics (Ryvarden, 2018, 2019, 2020; Decock and Ryvarden, 2020). Although 34 *Diplomitoporus* species are recorded in Index Fungorum,^4^ only the molecular sequences of *D. crustulinus* and *D. flavescens* are available in GenBank. Basidiomata of *D. crustulinus* and *D. flavescens* are shown in Figure 4.

*Specimens examined*: *Diplomitoporus crustulinus*. CHINA. Sichuan Province, Daocheng County, Yading Nature Reserve, on fallen branch of *Picea* sp., 11 August 2019, Cui 17394 (BJFC 034253); Jiulong County, on fallen branch of *Abies* sp., 12 September 2019, Cui 17690 (BJFC 034549). Yunnan Province, Shangri-La County, Pudacuo National Park, on fallen branch of *Picea* sp., 13 August 2019, Cui 1747*5* (BJFC 034334). *Diplomitoporus flavescens*. BELARUS. Brestskaya Voblasts, Belavezhskaya Pushcha National Park, on fallen trunk of *Pinus* sp., 18 October 2019, Dai 21020 (BJFC 032679). CHINA. Hebei Province, Zhuolu County, Xiaowutai Nature Reserve, on dead tree of *Pinus* sp., 9 September 2017, Dai 18096 (BJFC 025626), Dai 18097 (BJFC 025627). Jilin Province, Antu County, Changbaishan Nature Reserve, on fallen branch of *Pinus* sp., 20 September 2019, Dai 20846 (BJFC 032515). Sichuan Province, Xiangcheng County, on fallen branch of *Pinus* sp., 12 August 2019, Cui 1741*9* (BJFC 034278), Cui 17457 (BJFC 034316), Cui 17459 (BJFC 034318). Xizang Autonomous Region (Tibet), Linzhi, Bomi County, Bulang, on fallen branch of *Pinus yunnanensis*, 21 October 2021, Dai 23640 (BJFC 038212); on fallen trunk of *Pinus yunnanensis*, 21 October 2021, Dai 23650 (BJFC 038222); Chayu County, Cibagou Nature Reserve, on fallen branch of *Pinus densata*, 10 September 2020, Cui 18420 (BJFC 035281), Cui 1842*8* (BJFC 035289), Cui 18444 (BJFC 035305); Mangkang County, Jueba Mountain, on fallen branch of *Pinus densata*, 9 September 2020, Cui 18392 (BJFC 035253). Yunnan Province, Lanping County, Tongdian, Luoguqing, on fallen branch of Pinus yunnanensis, 3 September 2021, Dai 22798 (BJFC 037371).

Gloeoporellaceae B.K. Cui, Shun Liu & Y.C. Dai, fam. nov.

MycoBank: MB 840368

*Type genus*: *Gloeoporellus*.

*Diagnosis*: Basidiomata annual, resupinate to effused-reflexed, soft corky to corky when fresh, corky to fragile when dry. Hymenophores poroid. Hyphal system dimitic; generative hyphae with clamp connections, binding hyphae IKI–, CB+. Cystidia absent; cystidioles present. Basidiospores allantoid, colorless, thin-walled, smooth, IKI–, CB–. Causing a white rot.

*Genus*: *Gloeoporellus*.

*Gloeoporellus* Zmitr., Folia Cryptogamica Petropolitana 6: 85, 2018.

MycoBank: MB 827569

*Type species*: *Gloeoporellus merulinus*.

*Diagnosis*: Basidiomata annual, resupinate to effuse-reflexed, soft corky to corky when fresh, corky to fragile when dry. Pore surface buff-yellow, yellowish brown to apricot-orange when fresh, yellowish buff to orange-yellow when dry; pores round to angular. Context buff-yellow to orange-yellow, corky to fragile. Tubes concolorous with the pore surface, corky to fragile. Hyphal system dimitic; generative hyphae with clamp connections; binding hyphae IKI–, CB+. Cystidia absent; cystidioles present, tubular to fusoid, colorless, thin-walled, smooth. Basidia subclavate, colorless, thin-walled. Basidiospores allantoid to cylindrical, colorless, thin- to slightly thick-walled, smooth, IKI–, CB–. Causing a white rot.

**FIGURE 5:**
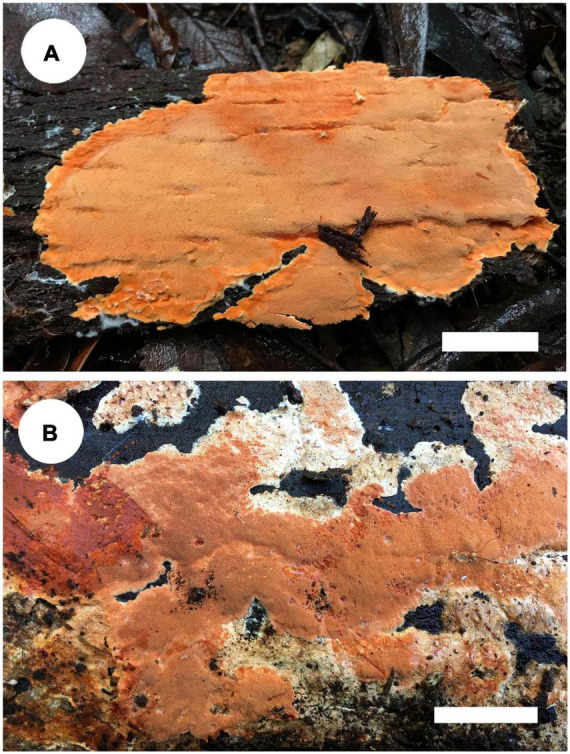
Basidiomata of *Gloeoporellus* species: *G. merulinus* (**A**: Cui 16724; **B**: Dai 18735). Scale bars: ***A***,***B*** = 2 cm.

*Notes*: *Tyromyces merulinus* was proposed by Cunningham (1965) as a new combination. This species distributes in Argentina, Australia, and New Zealand in the southern hemisphere, and the type locality is Tasmania, Australia (Cunningham, 1965). In Justo et al. (2017), *Tyromyces merulinus* cannot be placed with certainty in any of the recognized families. Zmitrovich (2018) presented the system of Polyporaceae and carried out the overview of the order Polyporales; *Gloeoporellus* was proposed as a new genus, with *Tyromyces merulinus* as type species. Zmitrovich (2018) and He et al. (2019) placed *Gloeoporellus* into Incrustoporiaceae Jülich, but without the support of phylogenetic analysis. Only one species, *Gloeoporellus merulinus*, is accepted in this genus now. Basidiomata of *G. merulinus* are shown in Figure 5.

*Specimens examined*: *Gloeoporellus merulinus*. AUSTRALIA. Tasmania, Mount Field Forest, close to Mount National Park, on rotten wood of *Nothofagus cunninghamii*, 14 May 2018, Dai 18734 (BJFC 027203); on rotten wood of Nothofagus sp., 14 May 2018, Dai 18735 (BJFC 027204); on living tree of Eucalyptus sp., 14 May 2018, Cui 16676 (BJFC 029975); Hobart, Mount Wellington, on rotten wood of Acacia sp., 13 May 2018, Cui 16629 (BJFC 029928); Timbs Track, on fallen trunk of Nothofagus sp., 14 May 2018, Cui 16650 (BJFC 029949), Cui 16668 (BJFC 029967); Arve River, Streamside Nature Reserve, on dead tree of *Eucalyptus* sp., 15 May 2018, Dai 18782 (BJFC 027250); on fallen trunk of Eucalyptus sp., 15 May 2018, Cui 16724 (BJFC 030023).

## Discussion

The Polyporales are a diverse group of Agaricomycetes, which have received extensive attention and studies. Some mycologists have attempted to adopt ribosomal RNA genes to study the phylogeny of Polyporales, but the results suggested that it is difficult to resolve the taxonomic structure of Polyporales (Larsson et al., 2004; Binder et al., 2005). Subsequently, protein-coding genes, including RPB1, RPB2, and TEF1, were applied to the phylogenetic study of Polyporales (Matheny et al., 2007; Binder et al., 2013; Zhao et al., 2015; Justo et al., 2017). To better verify the independent status of the two new families and provide more molecular data for future research, the phylogenetic analyses of Polyporales are inferred from the combined datasets of ITS + nLSU + RPB1 sequences (Figure 1) and ITS + nLSU + RPB1 + RPB2 + TEF1 sequences (Figure 2). The topological structures obtained from the phylogenetic analyses of the two sequence datasets are slightly different, probably due to the different gene fragments used in the phylogenetic analyses. The results showed that 27 lineages of Polyporales are recognized at the family level (Figures 1, 2), viz., Adustoporiaceae Audet, Cerrenaceae, Dacryobolaceae Jülich, Fibroporiaceae Audet, Fomitopsidaceae Jülich, Fragiliporiaceae, Gelatoporiaceae Miettinen, Justo and Hibbett, Grifolaceae Jülich, Hyphodermataceae Jülich, Incrustoporiaceae, Irpicaceae, Ischnodermataceae, Laetiporaceae Jülich, Laricifomitaceae Jülich, Meripilaceae Jülich, Meruliaceae, Panaceae, Phaeolaceae Jülich, Phanerochaetaceae Jülich, Podoscyphaceae, Polyporaceae, Pycnoporellaceae Audet, Sarcoporiaceae Audet, Sparassidaceae, Steccherinaceae, the climacocystis lineage (*Climacocystis* and *Diplomitoporus*) and *Gloeoporellus merulinus* (*Tyromyces merulinus*) could not be recognized in any existing families and they are proposed as two new families.

Binder et al. (2005) showed that *Climacocystis* nested inside the antrodia clade, but other analyses showed that *Climacocystis* grouped in the residual polyporoid clade (Miettinen et al., 2012; Binder et al., 2013; Justo et al., 2017). Justo et al. (2017) indicated that *Climacocystis* and *Diplomitoporus* grouped together with high support within the residual clade and could not be assigned to any recognized family of the Polyporales. Perhaps, previous studies lacked sufficient morphological features and molecular data to determine the family level of *Climacocystis* and *Diplomitoporus*, so their classifications at the family level were treated as incertae sedis (He et al., 2019). In the present study, *Climacocystis* and *Diplomitoporus* grouped together with high support within the residual clade (100% MP, 100% ML, 1.00 BPP; Figures 1, 2). This lineage has unique morphological characters and forms a well-supported clade. Phylogenetically, Climacocystaceae was closely related to the white-rot fungal families Hyphodermataceae, Meripilaceae, Podoscyphaceae, and Steccherinaceae (Figures 1, 2). Morphologically, Hyphodermataceae differs by having corticioid basidiomata, monomitic hyphal system, and thin-walled basidiospores; Meripilaceae differs in having monomitic or dimitic hyphal system without clamped generative hyphae; Podoscyphaceae differs by possessing mostly pileate basidiomata with smooth, ridged, or poroid hymenophore, dimitic or trimitic hyphal system, and thin-walled basidiospores; Steccherinaceae differs in possessing poroid or hydnoid hymenophore, dimitic hyphae system, mostly thin-walled, and rather small basidiospores (Reid, 1965; Parmasto, 1968; Jülich, 1981; Justo et al., 2017). Thus, a new family, Climacocystaceae, is proposed based on phylogenetic analyses and morphological characters. *Rickiopora* Westph., Tomšovskı, and Rajchenb. Were established by Westphalen et al. (2016) and related to *Climacocystis*. But in the current study, *Rickiopora* cannot be grouped with Climacocystaceae with high support (Figures 1, 2). *Hypochnicium* J. Erikss. and *Bulbillomyces* Jülich were grouped together and given an informal name as the hypochnicium clade, and the hypochnicium clade was closely related to the climacocystis clade without high support (Justo et al., 2017). In our current study, the hypochnicium clade is not closely related to the climacocystis clade (Figures 1, 2). Regarding the phylogenetic relationship between the climacocystis clade and related taxa, we found some differences between the current study and previous studies. This may be due to the difference in the number and composition of samples, gene fragments, and analysis methods used in the phylogenetic analysis. Nevertheless, all phylogenetic analyses support that the climacocystis clade cannot be placed in any recognized family and should be established as a new family.

Justo et al. (2017) revealed that *Tyromyces merulinus* is sister to Incrustoporiaceae, and this species cannot be assigned to a family within Polyporales. Subsequently, *Gloeoporellus* was proposed to accommodate *Tyromyces merulinus* (Zmitrovich, 2018) and was placed in the Incrustoporiaceae (Zmitrovich, 2018; He et al., 2019) without the verification of the phylogenetic analysis. In the present study, specimens of *Gloeoporellus merulinus* are grouped together with high support (100% MP, 100% ML, 1.00 BPP; Figures 1, 2), and are closely related to Fragiliporiaceae and Incrustoporiaceae without statistical support. Morphologically, Fragiliporiaceae resembles Gloeoporellaceae by having an annual growth habit, resupinate basidiomata, clamped generative hyphae, and thin-walled basidiospores. However, Fragiliporiaceae differs by its brittle basidiomata, grayish-buff to lavender pore surface when fresh, vinaceous gray to grayish brown when dry, larger pores, monomitic hyphal system, larger, and allantoid basidiospores (Zhao et al., 2015). Gloeoporellaceae and Incrustoporiaceae share poroid hymenophores, clamped generative hyphae and thin-walled basidiospores, but Incrustoporiaceae differs by having pileate, resupinate to effused-reflexed basidiomata, monomitic, dimitic to trimitic hyphal system, allantoid to ellipsoid or subglobose basidiospores, and tips of generative hyphae at tube mouths commonly with rose-thorn encrustations (Justo et al., 2017; Korhonen et al., 2018; Yuan et al., 2020). Thus, a new family, Gloeoporellaceae, is proposed to accommodate *Gloeoporellus merulinus*.

In the current study, the molecular clock analysis is executed to verify the taxonomic system for Polyporales with the estimated divergence time. The MCC tree (Figure 3) shows that the ancestor of Agaricales, Amylocorticiales, Cantharellales, Hymenochaetales, Hysterangiales, Phallales, Polyporales, Russulales, and Thelephorales split at about 120.6, 127.4, 243.29, 224.4, 93.89, 93.89, 136.53, 156.58, and 136.53 Mya, respectively; these data generally agree with previous studies (Chen et al., 2015; He et al., 2019; Wang et al., 2021; Ji et al., 2022). He et al. (2019) showed the mean of stem age of families within Agaricomycotina in a range of 27–259 Mya, among which the mean of stem age of six families in Polyporales ranged from 62 to 106 Mya. The current molecular clock analysis shows that the families in Polyporales diverged between 66.02 and 119.22 Mya (Figure 3 and Table 2), of which the Climacocystaceae occur in a mean stem age of 77.49 Mya and Gloeoporellaceae occur in a mean stem age of 88.06 Mya. According to the evolutionary divergence times, Climacocystaceae and Gloeoporellaceae could be recognized as independent families within Polyporales.

## Data availability statement

The datasets presented in this study can be found in online repositories. The names of the repository/repositories and accession number(s) can be found in the article/supplementary material.

## Author contributions

B-KC designed the experiment and conceived and supervised the work. SL, J-LZ, JS, Y-FS, Y-CD, and B-KC prepared the samples. SL and B-KC made the morphological examinations. SL performed the phylogenetic analyses. SL and J-LZ performed the molecular clock analysis. SL and JS performed the molecular sequencing. SL, J-LZ, JS, and B-KC wrote the manuscript. All authors contributed to the article and approved the submitted version.

## Footnotes

1
http://mafft.cbrc.jp/alignment/server/

2
http://tree.bio.ed.ac.uk/software/figtree/

3
http://tree.bio.ed.ac.uk/software/tracer/

4
http://www.indexfungorum.org
